# Periconception and First Trimester Diet Modifies Appetite, Hypothalamic Gene Expression, and Carcass Traits in Bulls

**DOI:** 10.3389/fgene.2021.720242

**Published:** 2021-09-03

**Authors:** Katrina J. Copping, Matthew J. Callaghan, Geert H. Geesink, Jessica R. Gugusheff, I. Caroline McMillen, Raymond J. Rodgers, Beverly S. Muhlhausler, Mini A. Vithayathil, Viv E. A. Perry

**Affiliations:** ^1^Robinson Research Institute, The University of Adelaide, Adelaide, SA, Australia; ^2^Ridley Agriproducts, Toowong, QLD, Australia; ^3^School of Rural and Environmental Science, University of New England, Armidale, NSW, Australia; ^4^Department of Food and Wine Science, FOODplus Research Centre, School of Agriculture, Food, and Wine, The University of Adelaide, Adelaide, SA, Australia; ^5^The Chancellery, University of Newcastle, Newcastle, NSW, Australia; ^6^Nutrition and Health Program, Health and Biosecurity Business Unit, CSIRO, Adelaide, SA, Australia

**Keywords:** appetite, beef cattle, carcass, fetal development, gene expression, hypothalamus

## Abstract

Nulliparous yearling beef heifers (*n*=360) were used to evaluate the effects of maternal dietary protein during the periconception and first trimester periods of gestation on postnatal growth, feedlot performance, carcass characteristics, and the expression of genes associated with appetite in the arcuate nucleus of their male progeny. Heifers were individually fed a diet of 1.18g crude protein (CP)/day High protein (HPeri) or 0.62g CP/day Low protein (LPeri) beginning 60days before conception. From 24 to 98days post-conception (dpc), half of each treatment group changed to the alternative post-conception diet and were fed 1.49g CP/day (HPost) or 0.88g CP/day (LPost) yielding four treatment groups in a 2×2 factorial design. From day 98 of gestation, heifers received a common diet until parturition. Calves were weaned at 183days and developed on pasture before feedlot entry. Bulls underwent a 70-day Residual Feed Intake (RFI) feedlot test commencing at 528days of age. Feedlot entry and final body weight (BW), feedlot average daily gain (ADG) and RFI were not different (*p*>0.05). Progeny of dams that had a change in diet (LPeri/HPost and HPeri/LPost) had 9% higher daily dry matter intake (DMI) during the RFI test (*p*<0.05) than progeny of dams that received low diet throughout both the peri-conception period and first trimester (LPeri/LPost). Further, mRNA expression of the appetite-stimulating agouti-related protein (AGRP) was increased in the arcuate nucleus of High Peri/LPost bulls (*p*<0.05). *Longissimus dorsi* muscle cross sectional area, carcass dressing percentage, and estimated retail beef yield (RBY) were all higher (*p*<0.05), and rump (P8) fat tended to be lower (*p*=0.07), for bulls from HPost dams despite no difference in carcass weight (*p*<0.05). This study is of commercial importance to the livestock industry as specific periods of maternal dietary supplementation may increase feed intake, enhance progeny muscling, and alter fat deposition leading to improvement in efficiency of meat production in beef cattle.

## Introduction

Protein is the most common limiting factor to production under Australian extensive pasture beef production systems (Bortolussi et al., 2005). In cattle, maternal dietary protein intake during pregnancy in heifers impacts fetal, as well as post-natal and adult growth, physiology, metabolism, and carcass traits of the offspring (Larson et al., 2009; Sullivan et al., 2009a; Micke et al., 2010a,b; Long et al., 2012; Copping et al., 2020). These bovine studies, and others in the ovine (Muhlhausler et al., 2006), illustrate that early gestation is an important period of development. Dams that experience periods of under nutrition in early, but not late gestation, may produce normal birthweight progeny (Copping et al., 2014) but post-natal performance may still be impacted through altered fetal development including changes in gene expression in tissues, such as the brain, muscle, and liver (Micke et al., 2011b; Alvarenga et al., 2016; Copping et al., 2020).

Changes in the development of the hypothalamic region of the brain that persist into adulthood may contribute to the programming of altered postnatal appetite regulation – this being the basis for studies into the fetal origins of human obesity (McMillen and Robinson, 2005; Ong and Muhlhausler, 2014). It is the case that fetal muscle and adipose tissue have a low priority for nutrient partitioning compared with vital organs such as the brain, particularly in the adolescent mother (Wallace et al., 2004), making them vulnerable to under nutrition during early and mid-gestation (Zhu et al., 2006; Du et al., 2010). Such nutritional perturbations can alter cell differentiation between muscle and adipose tissue during these critical periods and may modify body composition and carcass characteristics in the postnatal animal (Long et al., 2012; Du et al., 2013). Few studies, however, report on the impact of protein intake in cattle during the periconception period and early gestation on the subsequent progeny growth, appetite, or carcass characteristics (Micke et al., 2010a).

Our aim, therefore, was to evaluate the effect of dietary protein intake at levels experienced under Australian extensive pasture production systems in unsupplemented and supplemented 2-year old calving nulliparous heifers on progeny performance. Previously we have shown, in 3-year old calving nulliparous heifers, that nutrient intake during the first and second trimesters resulted in the programming of body weight (BW) and hot carcass weight (HCW) in the progeny in a sex-dependent manner (Micke et al., 2010a). In the current study, we hypothesise that maternal dietary protein supplementation during the peri-conception period and the first trimester in heifers calving at 2years of age, (an adolescent model) would increase post-natal performance and carcass characteristics whilst decreasing feed intake *via* effects upon gene expression in the arcuate nucleus in the male progeny.

## Materials and Methods

### Animals, Experimental Design, and Treatments

Use of animals and the procedures performed in this project were approved by University of South Australia IMVS Animal Ethics Committee (Australia), The University of Adelaide (Australia), and The University of New England (Australia) Animal Ethics Committees (Approval numbers: 18/11, S2012-249, and AEC14-037, respectively).

The purpose of this experiment was to determine the impact of maternal dietary protein during the peri-conception [PERI; −60–23days post-conception (dpc); conception being the day of artificial insemination (AI) and implantation being 18–22dpc; Wathes and Wooding, 1980; Spencer and Hansen, 2015] and first trimester (POST; 24–98dpc) periods in nulliparous heifers upon postnatal growth, appetite [as measured by Dry Matter Intake (DMI)], and carcass characteristics in the male progeny. The dietary protein levels reflected pasture conditions in Australian rangelands without (Low), and with (High), protein supplementation (Burns et al., 2010; Callaghan et al., 2020). The rations fed were as isocaloric as possible for ruminants receiving the forage component of their diet under group housing with a 1.9–2.1-fold difference in crude protein (CP) and a 1.1-fold difference in energy content between the high and low diets (Copping et al., 2018).

The study was a two-by-two factorial design. The study animals were the singleton male progeny of 2-year old heifers described previously (Copping et al., 2014) with management and dietary treatments reported in detail by Copping et al. (2018). Briefly, 360 nulliparous Santa Gertrudis (*Bos taurus*×*Bos indicus*) heifers underwent a 60-day acclimatisation during which time they were taught how to individually eat their daily ration from stalls. At 12months of age, 60days prior to AI, heifers were stratified by BW and randomly assigned to two equal PERI treatment groups, High and Low protein (HPeri and LPeri). Each heifer was individually fed a high [71 Mega joules (MJ) Metabolisable Energy (ME)/day and 1.18kg CP/day] or low protein diet (63MJ ME/day and 0.62kg CP/day; Supplementary Table S1; as fed basis). Heifers underwent a progesterone-based oestrous synchronisation programme commencing 10days prior to AI (Hernandez-Medrano et al., 2015) when all heifers were inseminated with frozen semen from one Santa Gertrudis bull. At 23dpc, half of each treatment group changed to an alternative post-conception (POST) treatment in the first trimester (Supplementary Table S1), High (HPost: 102MJ ME/day and 1.49kg CP/day), or Low (LPost: 98MJ ME/day and 0.88kg CP/day) giving rise to four treatment groups: [LPeri/LPost (LL), LPeri/HPost (LH), HPeri/LPost (HL), and HPeri/HPost (HH)].

Pregnancy was confirmed at 36dpc and fetal sex was determined at 60dpc by transrectal ultrasound (Copping et al., 2014). Non-pregnant heifers were removed from the trial. A sub-set of heifers (singleton fetus; *n*=46, 21 females, and 25 males) were euthanised at 98dpc (Copping et al., 2014). From 98dpc (the end of the first trimester of gestation), remaining heifers received a common diet formulated to provide growth of 0.5kg/d until parturition (79MJ ME/d and 0.92kg CP/d: as fed basis). Heifers were maintained in their groups in drylot pens with access to shade, water, and straw (5% CP) *ad libitum* and fed individually once daily until parturition. Sixty-four heifers completed the study and gave birth to 18 live singleton females (*n*: LL=3, LH=4, HL=4, and HH=7) and 43 live singleton bull progeny (*n*: LL=9, LH=10, HL=14, and HH=10). Two bull progeny were removed from the study after birth, due to poor mothering. An additional calf was subsequently found to be a cryptorchid and was excluded.

At parturition, individual feeding ceased. Progeny remained with their dams under extensive grazing conditions on native and improved pastures as one group until weaning (183.3±0.8days; age±SEM). A commercial molasses lick block containing 20% urea was offered at 200g/cow/day for the first 4months of lactation. Progeny were weighed at birth (Copping et al., 2014), then approximately monthly along with measurement of height at the cranial dorsal iliac spine (Micke et al., 2010a). During the post-weaning period, male progeny remained in one group under extensive grazing conditions on native rangeland and improved pastures prior to feedlot entry. No supplementation was offered for the first 4months post-weaning. Over the summer period from December, cereal hay [5.5% CP (DM basis), 57.4% Dry Matter Digestibility (DMD), and estimated ME content of 8.3MJ/kg DM] was offered *ad libitum* in racks. Following a large unseasonal rainfall event in February, this was withdrawn. The male progeny were left un-castrated to enable the assessment of their reproductive development (Copping et al., 2018).

### Milk Intake

The milk intake of the male progeny was measured using the weigh-suckle-weigh (WSW) protocol as previously described (Beal et al., 1990; Sullivan et al., 2009b) at 34, 65, 92, 125, 154, and 183days of post-natal life. Measurements were obtained by the summations of the changes in calf BW during three 20-min supervised sucklings undertaken after two 6-h intervals and a 12-h interval over a 24-h period (Sullivan et al., 2009b). The difference in pre- and post-suckling calf BW was considered to be the amount of milk consumed (Beal et al., 1990).

### Residual Feed Intake Test

Non-castrated singleton male progeny were transported to the “Tulimba” Research Feedlot, Kingstown, NSW (30°28'S, 151°11'E) at 507.3±0.8days (age±SEM) prior to the commencement of an Residual Feed Intake (RFI) test. Prior to testing, the animals underwent an adjustment period to the feeding environment and a grain diet (Arthur et al., 2004). One bull was removed following the pre-test introduction period after failing to adjust to the feeding environment. The bull remained on the same ration in an open bunk pen but was excluded from the study reported herein from 528days of age onwards. Remaining bulls (*n*: LL=8, LH=8, HL=13, and HH=10) underwent a standard 70-day RFI test (Exton, 2001). Briefly, during the RFI test, animals had *ad libitum* access to the standard high grain-content finishing ration (80% grain, 10% sorghum hay, and 5% protein pellets, plus a proprietary mixture of molasses, water, vitamin, and mineral additives; fresh weight basis). Samples of this ration from the start, midway through, and end of the RFI test were sent to a commercial feed evaluation service (NSW Department of Primary Industries Feed Testing Service, Wagga, NSW, Australia: https://www.dpi.nsw.gov.au/about-us/services/laboratory-services/feed-quality-service). The averaged content of the diet was 90% DM, 12% CP (DM basis), ether extract (EE) 4% of DM, 86% DMD, and estimated ME content of 13.5MJ/kg DM. Individual feed intake was measured using computerised automatic feeders (GrowSafe, Airdrie, Alberta, Canada) with each animal fitted with an electronic ear tag and individual feeding events recorded over the duration of the test period. Bulls were randomly allocated to two adjacent feedlot pens. The average age and BW (± SEM) at the commencement was 528.3±0.8days and 537.5±4.9kg, respectively. Individual animal BW was measured throughout the test period as per the standard RFI protocol (Exton, 2001). Metabolic mid-test BW (MMW) and average daily gain (ADG) were calculated from the regression of the animal’s fortnightly BW against day of test. RFI was calculated from the linear regression of average daily feed intake during the test (kg DM/day) against MMW (kg) and ADG (kg/day) with the residual being its RFI, as previously described (Arthur et al., 2001).

### Carcass Characteristics

Following the completion of the 70-day RFI test, the bulls were transported to a commercial abattoir, harvested, and then assessed for carcass traits. After slaughter, carcasses were weighed to give a HCW and prepared following standard AUS-MEAT procedure (Anon, 2007). Rump (P8) fat depth was recorded, before the sides of each carcass were chilled overnight. Carcasses were quartered the next morning between the 12 and 13th ribs. Carcass grading was undertaken by an accredited assessor. Traits measured were: *Longissimus dorsi* muscle (LM) area, AUS-MEAT marble score [0 (nil) to 6 (abundant)], fat color [0 (near white) to 4 (dark cream) by units of 1], and meat color [1A (pale pink) to 1C (dark pink); 2 (pale red); 3 (red)]. Estimated retail beef yield (RBY%) was calculated using the prediction equation Yield=64.8−(0.2 * P8)−(0.14 * EMA). Dressing percentage (dressing%) was calculated as HCW divided by the final non-fasted BW at the feedlot (Cafe et al., 2006). The whole brains of the animals were removed from the skull, frozen in isopentane over dry ice, and stored at −80°C for subsequent analysis of gene expression of hypothalamic appetite-regulating neuropeptides.

### Hypothalamic Gene Expression

The hypothalamic arcuate nucleus was isolated from the whole frozen brain collected using previously described methods (Muhlhausler et al., 2006). The frozen brains were initially sectioned using a medial sagittal incision to reveal the hypothalamus in each hemisphere (Warnes et al., 1998). A block containing the arcuate nucleus was then dissected from the brain bilaterally about 3mm from the midline, and dorsally about 6mm from the bottom of the brain using the caudal edge of the optic chiasma, and the rostral edge of the mammillary body as boundaries (Ong and Muhlhausler, 2011). Briefly, brains were initially sectioned using a medial sagittal incision to reveal the hypothalamus in each hemisphere. A block containing the arcuate nucleus was dissected from the brain bilaterally about 3mm from the midline, and dorsally about 6mm from the bottom of the brain using the caudal edge of the optic chiasma, and the rostral edge of the mammillary body as boundaries. RNA was extracted from the samples of arcuate nucleus using Trizol (Sigma-Aldrich Co., St. Louis, United States) and Qiagen RNAeasy Mini kit according to the manufacturer’s instructions (Qiagen Pty Ltd., Doncaster, Australia). RNA integrity was confirmed by agarose gel electrophoresis and RNA concentration measured using a Nanodrop (Thermo Scientific, DE, United States). Total RNA (~2μg) was then reversed transcribed into cDNA using Superscript III reverse transcriptase (Invitrogen Australia Pty Ltd., Mount Waverley, Australia) with random hexamers. A No Amplification Control (NAC), in which Superscript III was replaced with molecular grade water, was also prepared for each sample to confirm the absence of genomic DNA contamination in each sample.

The mRNA expression of the appetite stimulating [Agouti-Related Protein (*AGRP*), Neuropeptide Y (*NPY*)] and appetite-inhibiting [Proopiomelanocortin (*POMC*), Cocaine and Amphetamine-Regulated Transcript (*CART*)] neuropeptides and leptin receptor (*OBRb*) in the arcuate nucleus was determined using quantitative RT-PCR (qRT-PCR) using SYBR green in the Viia7 System (Applied Biosystems, Foster City, CA, United States). A constant amount of cDNA (1μl) was used for each qRT-PCR measurement and at least three technical replicates were performed for each gene. Each qRT-PCR reaction well (10μl total volume) contained: 5μl iTaq™ SYBR® Green supermix 2x (Bio-Rad Laboratories, Hercules, CA, United States); 1μl of forward and reverse primer giving a final concentration of 600 or 900nM, 2μl of molecular grade H_2_O, and 1.0μl of a 50ng/μl dilution of the stock template. The cycling conditions consisted of a hold stage of 95°C for 5min followed by a PCR stage, which included 40cycles of 95°C for 20s, 55°C for 20s, and 72°C for 40s. A melt curve stage was included (95°C for 15s, 60°C for 1min, and 95°C for 15s) to confirm the specificity of the reaction. At the end of each run, dissociation melt curves were obtained. Three quality controls as well as two negative controls for each primer set were included on each 96-well plate in order to verify inter-plate consistency, and the inter-plate CV was <5% for all experiments.

Primer sequences are shown in Table 1. The abundance of each mRNA transcript was quantified relative to two normaliser genes (selected using the BestKeeper program), β-actin (Quantitect primer assay, Qiagen Australia, Doncaster, Vic, Australia), and eukaryotic translation initiation factor 3 subunit K (*EIF3K*, Forward: 5'-TGA CAG ACA GCC AGC TAA AGG TGT-3', Reverse: 5'-TCT TCT CCA CGA TGT TCT TGG GCT-3') using the Applied Biosystems Data Assist software (Applied Biosystems, Foster City, CA, United States). This software allows the expression of each target gene to be measured against the mean normalised expression of the two normalisers. All primer sequences had been previously published for use in bovine tissues (Kobayashi et al., 2004; Ainu Husna et al., 2012; Perkins et al., 2014). The amplicons were sequenced prior to the experiment to ensure the authenticity of the DNA product. For the qRT-PCR measurements, the primer concentrations were consistent for all genes and the amplification efficiency of all primers was 0.995–0.999.

**Table 1:** Sequences of primers used for real-time PCR quantification of gene expression.

Gene^1^	Forward primer (5'–3')	Reverse primer (5'–3')
*NPY*	5'-TAG CGG AGC GTG ATT GCC CG-3'	5' -GGG GGT GTC CGG AGC AGG TT-3’
*AGRP*	5'-GGG CAC CCC TCT TGT AGA GCC-3'	5'-GGC CCA CAC GTG ACT GCT TCC-3'
*POMC*	5'-GCC GCT GAA CAT CCT CGC CC-3’	5'-CTC CAG GCA CCA ACC ACG CA-3'
*OBRb*	5'-GGCCTGGATGAACTTTTGAA-3'	5'-TGTGAGCAACTGTCCTGGAG-3'
*CART*	5'-ACG CGT CCG GTT TCA GCA CCA T-3	5'-CTTGACAGA TGA CAT CAC AACC-3'

1 NPY, neuropeptide Y; AGRP, agouti-related protein; POMC, proopiomelanocortin; OBRb, leptin receptor; and CART, cocaine and amphetamine-regulated transcript.

### Statistical Analysis

Two-way ANOVA (STATA/IC 13.0, StataCorp, College Station, Texas, United States), with *post hoc* Tukey-Kramer test as required, was used to interpret the effects of maternal nutrition treatment group during PERI and POST treatment periods and their interaction term on BW, ADG, height, DMI, RFI, HCW, LM area, P8 Fat depth, fat color, AUS-MEAT meat color and marble score, dressing %, RBY%, and hypothalamic gene expression. All traits were analysed as if continuous with meat color coded: 1A=1, 1B=1.3, 1C=1.7, 2=2, and 3=3 (Herd et al., 2018). Feedlot pen influenced ADG and thus was included as a co-variate along with animal age. Additionally, to investigate the interactions between maternal diet and time on BW, height, and milk intake, linear mixed-effects models were performed, adjusting for repeated measures over time for each of the bulls. An autoregressive one covariance structure was used. *Post-hoc* comparisons were made of the differences of least squares means as required. The statistical software used was SAS 9.3 (SAS Institute Inc., Cary, NC, United States). Statistical significance was accepted at *p*<0.05, and a tendency at *p*<0.10.

## Results

### Pre-weaning and Post-weaning Progeny Performance

**Figure 1:**
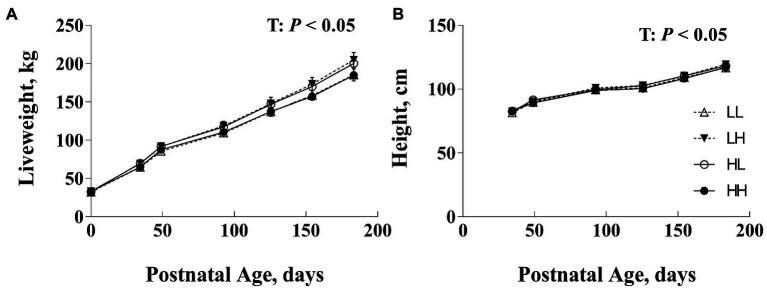
Postnatal body weight (BW; **A**) and hip height **(B)** of bulls following exposure to maternal diets low (L) and/or high (H) in protein during the peri-conception (PERI: −60–23dpc) and first trimester (POST; 24–98dpc) periods of gestation^1^. T, time effects (*p*<0.05). No significant differences between maternal diet groups overall or among groups within age (*p*>0.05). ^1^LL, Low protein maternal diet during both PERI and POST diet periods. LH, Low protein maternal diet during PERI and High protein during POST diet periods. HL, High protein maternal diet during PERI and Low protein during POST diet periods. HH, High protein maternal during both PERI and POST diet periods.

At birth, there was no difference in progeny birth BW (Copping et al., 2014). Progeny performance at weaning and post-weaning prior to RFI test are presented in Table 2. Post-natal progeny BW, ADG, and height at weaning tended to vary with an interaction between PERI and POST diet (*p*<0.10), but there were no differences between diet groups (*p*>0.10). Post-natal progeny BW, ADG, and height prior to RFI test, did not differ due to maternal nutrition (*p*>0.10; Table 2). There was no effect of gestation length on progeny BW, ADG, and height (*p*>0.10). When data from birth until weaning was considered, BW and height changed with time (*p*<0.05; Figure 1) but there were no interaction effects, nor effects of the level of maternal nutrition during the PERI or POST periods (*p*>0.10).

**Table 2:** Liveweight (BW), height, and ADG of bulls at weaning and post-weaning prior to commencement of residual feed intake test, following exposure to maternal diets low or high in protein during the peri-conception and first trimester periods of gestation^1^.

	Treatment						
PERI	Low		High		*p* value		
POST	Low	High	Low	High	PERI	POST	PERI*POST^2^
Item							
*Weaning, 183days of age*							
*n*	8	8	14	10			
ADG^3^, kg/d	0.84±0.04	0.93±0.05	0.90±0.03	0.84±0.03	0.657	0.555	0.069
Height, cm	116.5±0.9	119.5±1.2	118.1±1.0	117.0±0.8	0.645	0.316	0.075
BW, kg	185.1±7.7	204.7±9.8	200.1±5.2	184.7±4.4	0.619	0.524	0.100
*Post-weaning, 183–520days of age*							
*n*	8	8	14	10			
ADG^3^, kg/d	1.00±0.03	0.94±0.0.3	0.95±0.02	0.95±0.03	0.592	0.138	0.415
Height at 520days, cm	137.1±1.3	136.9±1.6	139.0±1.3	136.3±1.3	0.662	0.334	0.432
BW at 520days, kg	520.8±6.5	520.0±14.1	519.3±6.5	505.2±9.5	0.418	0.388	0.423

No significant differences between treatments (*p*>0.05).

Values are unadjusted mean±SEM.

1 Dams were individually fed diets Low or High in protein during the periconception (PERI; −60–23days post-conception) and first trimester (POST; 24–98 dpc) periods of gestation.

2 PERI*POST=Interaction term (2×2 factorial design). Interactions (*p*<0.10) explored by *post hoc* test as required.

3 ADG, average daily gain (kg/d).

### Milk Intake of Bull Progeny

**Figure 2:**
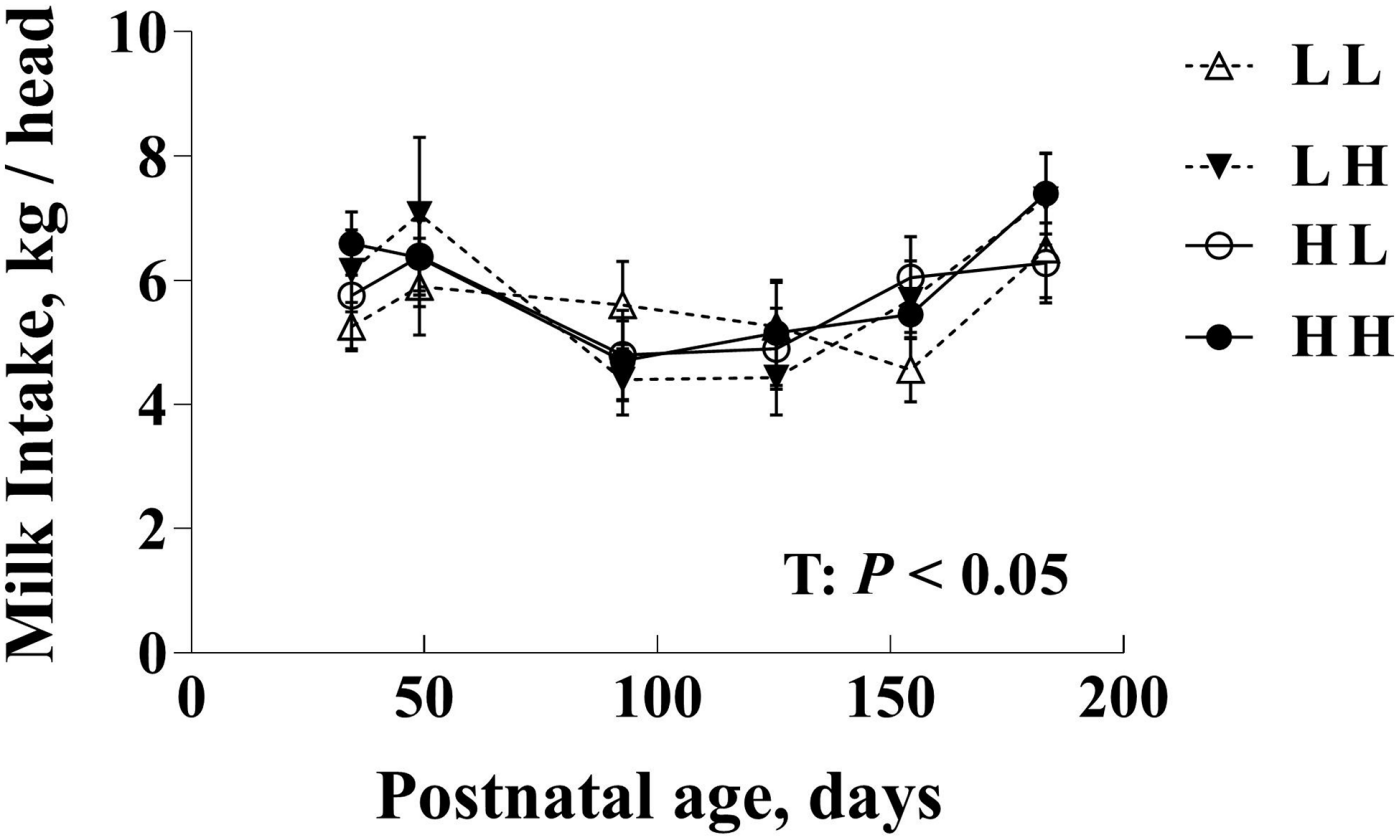
Daily milk intake (kg/head) in bull calves prior to weaning following exposure to maternal diets low (L) and/or high (H) in protein during the peri-conception (PERI; −60–23dpc) and first trimester (POST; 24–98dpc) periods of gestation^1^. T: Time effects (*p*<0.05). No significant differences between maternal diet groups overall or among groups within age (*p*>0.05). ^1^LL, Low protein maternal diet during both PERI and POST diet periods. LH, Low protein maternal diet during PERI and High protein during POST diet periods. HL, High protein maternal diet during PERI and Low protein during POST diet periods. HH, High protein maternal during both PERI and POST diet periods.

Milk intake varied with time (*p*<0.05) but did not differ due to maternal diet either overall or within each time point (*p*>0.10; Figure 2).

### Feedlot Growth and Efficiency

Initial feedlot entry BW and final BW, ADG, DMI, and RFI data are summarized in Table 3. There were no significant differences (*p*>0.05) in BW neither at the start nor at the end of the feedlot period between bulls from the different maternal nutrition treatment groups. Feed intake varied with a significant interaction (*p*<0.05) between maternal PERI and POST diet. Bulls whose dams had a change in diet at the end of the PERI diet period from High protein to Low protein and *vice versa* (HL and LH progeny) had 9% higher daily DMI on test (*p*<0.05) than those whose dams received a constant low protein diet throughout both the PERI and POST diet period (LL progeny). A similar pattern was apparent in feedlot growth rate (ADG) and RFI during the feedlot period but the differences were not statistically significant (*p*>0.10).

**Table 3:** Performance of bulls during 70days residual feed intake test following exposure to maternal diets low or high in protein during the peri-conception and first trimester periods of gestation^1^.

Treatment							
PERI	Low		High		*p* value		
POST	Low	High	Low	High	PERI	POST	PERI*POST^2^
Item							
*n*	8	8	13	10			
Initial BW at 528days of age, kg	532.8±6.3	545.1±16.5	541.5±7.6	530.2±9.3	0.774	0.917	0.296
Final BW at 598days of age, kg	647.4±15.5	678.6±19.1	676.6±10.8	652.3±11.4	0.961	0.870	0.125
ADG^3^ on RFI^4^ test, kg/d	1.66±0.19	1.94±0.08	1.96±0.07	1.77±0.08	0.594	0.873	0.128
DMI on RFI test, kg/d	12.4^ad^ ±0.4	13.6^bc^ ±0.4	13.6^bc^ ±0.3	12.7^cd^ ±0.4	0.836	0.809	0.032
RFI, kg/d	−0.12±0.22	0.18±0.26	0.15±0.16	−0.10±0.21	0.966	0.911	0.297

a,b,c,d Within a row, means without a common subscript differ at *p*<0.05 for treatment.

Values are unadjusted mean±SEM.

1 Dams were individually fed diets Low or High in protein during the periconception (PERI; −60–23dpc) and first trimester (POST; 24–98dpc) periods of gestation.

2 PERI*POST=Interaction term (2×2 factorial design). Interactions (*p*<0.10) explored by *post hoc* test as required.

3 ADG, average daily gain (kg/d).

4 RFI, residual feed intake.

### Carcass Measures

Carcass measures are presented in Table 4. Hot carcass weight (HCW), AUS-MEAT marble score, AUS-MEAT meat color, or fat color did not differ (*p*>0.10) as a result of PERI and POST maternal protein intake, nor were there any interaction effects. Dressing % increased (*p*<0.05) in those animals whose dams received the HPost diet (24–98dpc), with HPost (HH+LH) bulls having a 1.5 percentage point increase over LPost (LL+HL) bulls (54.8±0.4 *vs.* 53.3±0.4%, respectively; *p*=0.030). Rump (P8) fat depth did not differ as the result of maternal nutritional treatment, although those animals born to dams receiving HPost diet (HH+LH) tended to have decreased P8 fat depth compared to LPost (LL+HL) bulls (16.0±0.9 *vs.* 18.2±1.1mm, respectively; *p*=0.065). When adjusted for HCW, there was no difference in fatness, although animals born to dams receiving the HPost (HH+LH) still tended to be leaner (*p*=0.07).

**Table 4:** Carcass characteristics of bulls at slaughter at 598days of age following exposure to maternal diets low or high in protein during the peri-conception and first trimester periods of gestation^1^.

	Treatment						
PERI	Low		High		*p* value		
POST	Low	High	Low	High	PERI	POST	PERI*POST^2^
Item							
*n*	8	8	13	10			
P8 Fat^3^,mm	17.0±1.8	15.0±0.3	19.7±1.6	16.2±1.1	0.233	0.065	0.921
LMA^4^, cm^2^	80.3±3.3	89.5±2.5	81.7±1.9	86.8±4.4	0.910	0.027	0.318
Dressing^5^,%	53.2±0.7	54.9±0.6	53.4±0.5	54.8±0.7	0.918	0.030	0.620
RBY^6^, %	72.6±0.4	74.3±0.4	72.3±0.5	73.7±0.7	0.466	0.007	0.387
HCW^7^, kg	344.3±8.9	373.1±13.1	361.3±6.7	357.3±8.0	0.960	0.320	0.130
Meat color code	1.76±0.08	1.78±0.05	1.88±0.04	1.81±0.07	0.210	0.978	0.470
Fat color code	1.50±0.27	1.0±0.19	1.15±0.10	1.1±0.18	0.502	0.214	0.256
Marble score	0.13±0.13	0.13±0.13	0.15±0.10	0.50±0.17	0.131	0.231	0.416

Values are unadjusted mean±SEM.

1 Dams were individually fed diets Low or High in protein during the periconception (PERI; −60–23dpc) and first trimester (POST; 24–98dpc) periods of gestation.

2 PERI*POST=Interaction term (2×2 factorial design). Interactions (*p*<0.10) explored by *post hoc* test as required.

3 P8 Fat, P8 Fat depth.

4 LMA, LM area.

5 Dressing=Dressing %=HCW/BW.

6 RBY=Retail Beef Yield %=64.8−(0.2×P8)−(0.14×LMA).

7 HCW, hot carcass weight.

*Longissimus dorsi* muscle area was larger in those animals whose dams received a high protein diet during the first trimester (24–98dpc), with HPost (HH+LH) bulls showing a 6.9cm^2^ increase in LM area over LPost (LL+HL) bulls (88.0±2.6 *vs.* 81.1±0.9cm^2^, respectively; *p*=0.027). This difference was significant both with and without adjustment for HCW (*p*<0.05). RBY % was significantly greater in those animals whose dams received a high protein diet during the first trimester (24–98 dpc) with HPost (HH+LH) bulls having a 1.5 percentage point increase in RBY% over LPost (LL+HL) bulls (74.0±0.4 *vs.* 72.4±0.4%, respectively; *p*=0.007). This was despite there being no significant difference in BW at slaughter, or in HCW. The inclusion of progeny birth BW or maternal BW at calving as a covariate in the statistical model (Robinson et al., 2013) did not alter the significance of the effects of maternal diet on the carcass traits described above.

### Hypothalamic Neuropeptide mRNA Expression

**Figure 3:**
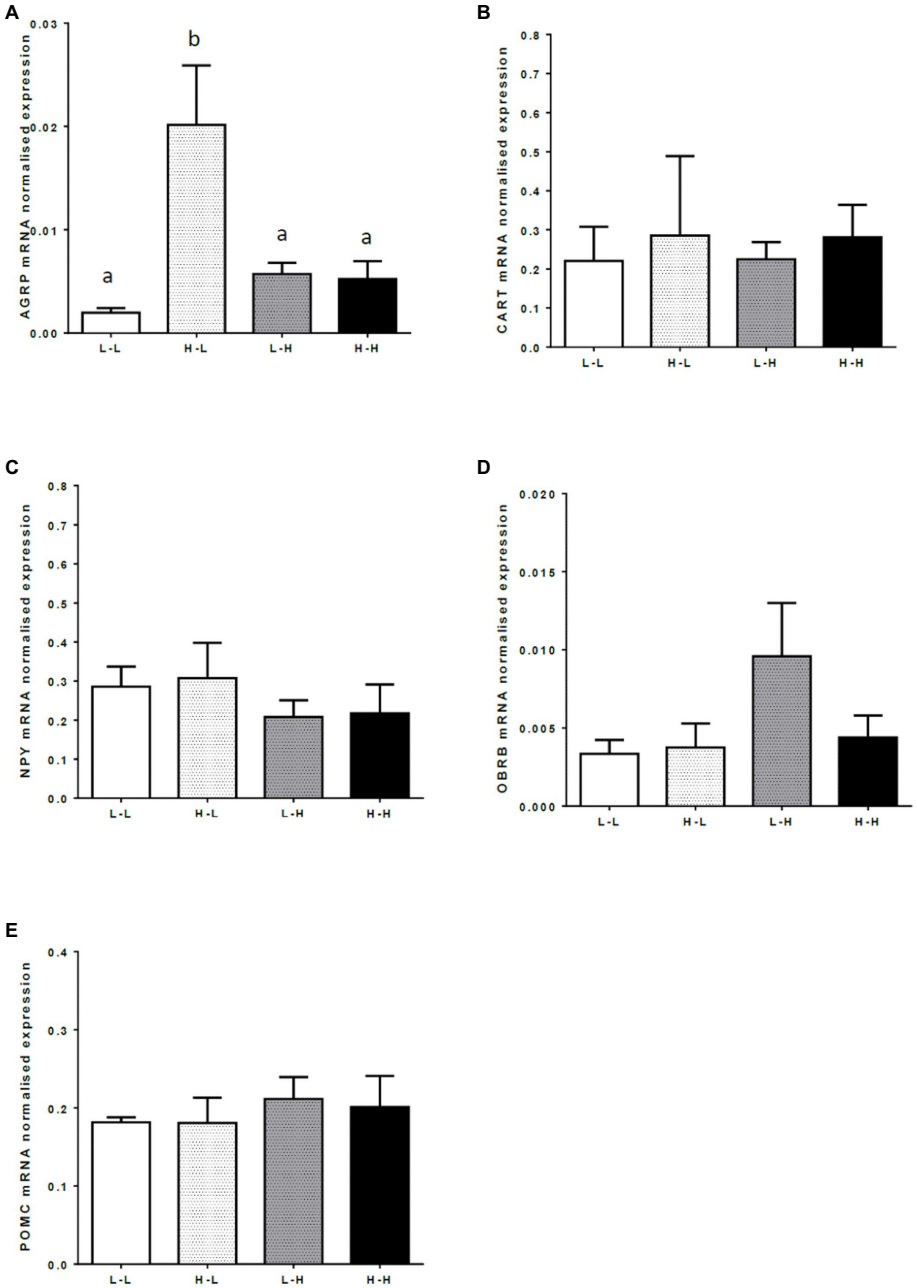
Mean normalized hypothalamic mRNA expression of **(A)** Agouti-Related Protein (*ARGP*); **(B)** Cocaine and Amphetamine-Regulated Transcript (*CART*); **(C)** Neuropeptide Y (*NPY*); **(D)** Leptin receptor (*OBRB*); and **(E)** Proopiomelanocortin (*POMC*) in the arcuate nucleus from 20-month old bulls exposed to maternal diets low (L) and/or high (H) in protein during the peri-conception (PERI; −60–23dpc) or first trimester (POST; 24–98dpc) periods of gestation^1^. **(A)** ^a,b^Means without a common letter differ (*p*<0.05). **(B–E)** No significant differences between maternal diet groups (*p*>0.05). ^1^LL, Low protein maternal diet during both PERI and POST diet periods. LH, Low protein maternal diet during PERI and High protein during POST diet periods. HL, High protein maternal diet during PERI and Low protein during POST diet periods. HH, High protein maternal during both PERI and POST diet periods.

There was an interaction between the effects of PERI and POST maternal diet on mRNA expression of the appetite-stimulating neuropeptide, *AGRP*, in the hypothalamic arcuate nucleus (*p*<0.05). Low protein in the POST diet period increased *AGRP* mRNA expression in progeny from dams fed high protein in the PERI diet period (HL progeny) but a low protein diet in the POST period had no effect on *AGRP* mRNA expression in progeny from dams fed a low protein diet during the PERI diet period (LL progeny; Figure 3A; *p*<0.05). There was no effect of maternal diet on mRNA expression of *CART*, *POMC*, *NPY*, or *OBRb* in the hypothalamic arcuate nucleus of the finished adult bull progeny (Figures 3B–E; *p*>0.05).

## Discussion

This study has demonstrated that dietary protein supplementation during the peri-conception period (PERI; −60–23dpc) and first trimester (POST; 24–98dpc) in beef heifers calving at 2-year of age altered genes associated with appetite in the arcuate nucleus, appetite, and carcass characteristics in their 20-month old non-castrated singleton male progeny. The mRNA expression of the appetite-stimulating *AGRP* was increased in the arcuate nucleus of bulls from HPeri/LPost dams. Concomitantly, the progeny of dams that had a change in diet between the PERI and POST periods had higher DMI on growth performance test than those progeny from the of dams that received Low diet throughout both the diet periods. Furthermore, there was an increase in RBY% related to an increased LM area and tendency for reduced P8 fat depth in the HPost cohort compared to LPost. The period of nutritional intervention during the first trimester corresponds to the period of primary myogenesis and the start of secondary myogenesis, which concludes at 180dpc (Stickland, 1978; Bonnet et al., 2010), with myofibre numbers fixed at this time (Bonnet et al., 2010). Protein supplementation during this critical period of fetal development may therefore be an important factor influencing carcass characteristics in the progeny.

In the present study, maternal dietary protein intake during either the PERI (−60–23dpc) or POST (24–98dpc) periods had no effect on birth BW (Copping et al., 2014), post-natal growth (pre and post-weaning BW, ADG, and height) nor upon feedlot performance or HCW at slaughter. This contrasts with the differences in measures of fetal growth observed between 36 and 98dpc in this cohort (Copping et al., 2014, 2020). These results for progeny BW, ADG, and HCW are however consistent with Long et al. (2010) who reported that global nutritional restriction (55% of NRC recommendation *vs.* 100% of NRC recommendation) during early pregnancy (32–83dpc) in 2-year old heifers did not affect birth BW or postnatal growth (ADG) in steer progeny. The results for progeny BW however, differed from that of Long et al. (2010) and from our previous findings in steer progeny from 3-year old heifers (Micke et al., 2010a). Long et al. (2010) reported that 16-month steers from the nutrient restricted dams were heavier at the beginning of the finishing period prior to slaughter and tended to be heavier at slaughter at 21-month, although HCW was not different. Micke et al. (2010a) reported that a low protein diet during the first trimester (0–93dpc) resulted in heavier post-natal BW at feedlot entry at 18-month but did not influence HCW in the steer progeny at 23-month. In contrast, in the heifer progeny (Micke et al., 2010a), those exposed to the low protein diet during the first trimester had a lower HCW at slaughter. These inconsistencies among studies suggest that the timing of the nutritional insult as well as progeny sex is critical in determining the effect upon offspring development, which is consistent to observations in humans and ruminants (Heijmans et al., 2008; Sinclair et al., 2016).

### Maternal Nutrition and Offspring Carcass Characteristics

Development of skeletal muscle *in utero* is crucial to final muscle definition as the number of muscle fibres does not alter after birth, in contrast to fibre size (Stickland, 1978; Zhu et al., 2004). As discussed above, during gestation, the delivery of nutrients to heart or brain is a higher priority than organs less vital for survival such as skeletal muscle (Reynolds and Caton, 2012) making it more vulnerable to maternal nutritional perturbation (Close and Pettigrew, 1990; Zhu et al., 2006). In the present study, increased protein intake in the dam from 24 to 98 dpc (first trimester) resulted in a higher LM area at slaughter. This period of nutritional intervention corresponded to the period of primary myogenesis and early secondary myogenesis (Russell and Oteruelo, 1981; Du et al., 2010) – when the maximum numbers of fetal muscle fibres develop in the bovine (Bonnet et al., 2010). Bulls born to dams fed a higher protein diet in the first trimester also showed improvement in RBY% compared with those born to dams fed a lower protein diet during this period. Differences in LM area are known to be reflected in RBY (McKiernan et al., 2009) and the observed larger LM area, combined with a tendency to be leaner, likely contributed to the increased RBY% in these bulls. Interestingly, the effects of HPost maternal diet on LM area remained when LM area was adjusted for HCW, suggesting that the HPost bulls had increased muscularity independent of overall BW.

The tendency for the progeny of the bulls born to dams exposed to a low protein diet during the first trimester to be fatter is consistent with previous reports of progeny of nutrient restricted dams being predisposed to adiposity (Micke et al., 2011a; Long et al., 2012). Interestingly, in fetal liver tissue at 98dpc, the low protein maternal diet altered expression of transcription factors regulating a number of hepatic genes (Copping et al., 2020) that effect liver metabolism and function including an increase in the insulin-independent glucose transporter *GLUT1* and a decrease in *PPARα*, which is integral to lipid and carbohydrate homeostasis (Brown and Plutzky, 2007). This is in agreement with previously published data following moderate global restriction during the first 50days of gestation in the bovine model that showed altered expression of hepatic genes associated with metabolic pathways and pathways associated with tissue accretion and function (Crouse et al., 2019).

As proposed by others (McMillen and Robinson, 2005; McMillen et al., 2008), increased adipogenesis may be a survival advantage under poor post-natal nutritional conditions. Adipogenesis in beef cattle starts to occur before mid-gestation (Bonnet et al., 2010). Adipocytes are reported to be seen first in visceral fat depots as early as 80dpc, sequentially followed by detection in subcutaneous and intermuscular depots, and finally, in intramuscular depots by 180dpc (Taga et al., 2011; Du et al., 2013). The 24–98dpc nutritional supplementation window (POST diet) in the current study corresponds to the very start of the window for initiation of adipocyte formation in the visceral and subcutaneous depots. The tendency for the progeny of dams fed a high protein diet during the first trimester to be leaner, have improved dressing% and higher RBY%, in the absence of differences in either pre-slaughter BW or HCW, suggests a shift in fat deposition that may improve carcass value.

We did not observe any differences in AUS-MEAT marble score between treatment groups, and scores were low overall. Previous literature reports differences in adiposity based on gender and castrate status (Berg and Butterfield, 1981) and the low marbling scores observed are likely to be related to the non-castrated status of the animals and their genetic type. Furthermore, the AUS-MEAT marble score reported is not a continuous scale, which may have also decreased our ability to detect differences between treatments. However, the similarity in marble score is supported by the laboratory assessment of intramuscular fat in samples from this cohort (Alvarenga et al., 2016). Intramuscular adipocyte formation is thought to predominately occur during late gestation through to about 250days postnatal (Taga et al., 2011), which was outside the supplementation window. In the current study, peri-conception diet was found to alter tenderness of the semitendinosus muscle, but not of the LM (Alvarenga et al., 2016). The lack of effect of the PERI diet upon any of the measures of carcass characteristics reported here is not unexpected given that evidence suggests that, although myocytes, adipocytes, and fibroblasts originate from a common progenitor during early embryogenesis (Du et al., 2013), the majority of myogenesis and adipogenesis occurs outside the peri-conception window (−60–23dpc; Du et al., 2010).

In other species, including the pig and sheep, similar changes in body composition to those observed in the current study such as increased adiposity and decreased muscle mass have also been reported. In one study, a smaller loin area and increased fatness were found in the offspring of sows fed a low-protein diet during gestation (Rehfeldt et al., 2011). Similarly, lambs from ewes that experienced nutrient restriction during mid-gestation were fatter and had a lower lean–to–fat ratio compared to progeny from non-restricted dams (Zhu et al., 2006). However, previous studies evaluating maternal nutrition effects on the carcass characteristics of bovine offspring have produced variable results (Greenwood et al., 2009; Larson et al., 2009; Underwood et al., 2010; Micke et al., 2010a, 2011b; Long et al., 2012; Mohrhauser et al., 2015). This has been attributed to differences between studies in the timing and length of the intervention period, the degree of nutrient restriction, dam age and parity, the specific nutrient evaluated, and the sample size or the sex evaluated (Robinson et al., 2013). The current study differs from these previous investigations (Larson et al., 2009; Micke et al., 2010a; Long et al., 2012; Mohrhauser et al., 2015) in the timing and length of the feeding intervention as feed intake was individually controlled from prior to conception through to parturition and in the use of yearling rather than 2-yo primiparous heifers.

### Maternal Nutrition and Offspring Feed Intake and Efficiency

Altered levels of nutrition during fetal and early postnatal development have been shown to influence offspring appetite and body composition (Ong and Muhlhausler, 2014). Exposure to both over and under-nutrition before birth has been reported to permanently change appetite regulation in humans, rodents, and sheep (Muhlhausler et al., 2006; Muhlhausler, 2007). Circulating hormones such as leptin and ghrelin provide feedback on an animal’s nutritional status and energy balance regulating the neural network in the hypothalamus that controls feed intake and appetite (Muhlhausler et al., 2006; Muhlhausler, 2007; Ainu Husna et al., 2012). In ruminants, these neural pathways develop early in pregnancy and have been reported to be susceptible to maternal nutritional perturbations (Muhlhausler et al., 2006).

Previously published data from the current study have shown that restricted protein intake between 24 and 98dpc resulted in sex-specific asymmetric development by 98dpc in a subset of fetuses; a characteristic of interuterine growth restriction (IUGR; Copping et al., 2020). The LPost diet increased the brain: fetal weight ratio indicating a brain sparing effect where brain growth is maintained at the expense of the development of other organs as previously reported in sheep studies (McMillen et al., 2001; Morrison, 2008). Here, we report observed changes to the expression of appetite regulating genes in the arcuate nucleus similar to that previously observed in the sheep (Atkinson and Adams, 1988). In particular, the appetite-stimulating neuropeptide, *AGRP*, was increased in the HPeri/LPost progeny and was associated with increased DMI in adulthood.

Total milk intake assessed during the pre-weaning period was unaffected in these male progeny. This is dissimilar to the effect we observed in male progeny of 3-year old calving heifers where milk intake was greater in those who had experienced low protein in the first trimester (Micke et al., 2015) accompanied by increased rates of ADG. Reports of altered milk intake in lambs following maternal diet perturbations in several studies have been restricted to measures in early postnatal life with effects not measured, or not persisting, beyond the first few weeks of life (Muhlhausler et al., 2006; De Blasio et al., 2007). It is possible, that differences in feed intake only emerged, or became more pronounced, in the post-weaning phase, and when factors such as dam milk production, maternal behaviour, and maternal-calf interaction would no longer have an influence (Miguel-Pacheco et al., 2019). A study in goats showed that maternal feed restriction during late pregnancy modified feeding behaviour in a small number of artificially reared female offspring for up to 2-year of age (Laporte-Broux et al., 2012) but the naturally reared male cohort exhibited no differences in milk intake or feeding behaviour in early neonatal life (Laporte-Broux et al., 2011).

The observed increase in DMI during the feedlot period in progeny from dams that had a change in diet between PERI and POST periods of gestation may have the potential to influence production efficiency, since feed is a major cost in livestock production, particularly during a feedlot finishing phase. RFI, a method to assess feed efficiency in the bovine (Cafe et al., 2014), did not differ between the groups. It is possible the relatively small number of animals studied meant there was insufficient power to detect anything but large differences in RFI (Herd et al., 2018). There are limited bovine studies that have assessed DMI and RFI in progeny whose dams experienced either restricted or excessive nutrition during pregnancy. Summers et al. (2015a) reported that RFI, as measured *via* a GrowSafe system, was improved in progeny born to dams that received isonitrogenous and isocaloric supplements with varying levels of rumen undegradable protein in late gestation (approx. 142–242dpc; Summers et al., 2015b). DMI, however, was greater in calves from unsupplemented dams in this same study. However, the period of nutritional intervention occurred much later in gestation than in the current study, and it is therefore difficult to compare these two studies.

Previous studies suggest that alterations to the plane of nutrition during embryonic and fetal development can induce adaptive physiological and epigenetic changes, which may have persistent consequences for the subsequent growth and development of the fetus and progeny, including potential adaptations to the regulation of appetite (Zhang et al., 2010, 2011; George et al., 2012). This may underlie the increase in DMI following a switch in maternal protein intake between the peri- and post-conceptional periods. In the case of the HL progeny, the elevated hypothalamic gene expression for the appetite stimulating neuropeptide, *AGRP* may have contributed to the observed higher DMI in this group, as AGRP stimulates food intake in ruminants (Wagner et al., 2004). In the LH progeny, it is possible that epigenetic modifications contributed to altered responsiveness of appetite regulatory genes to the prevailing nutritional supply, and therefore the increased food intake observed, in the absence of any changes in mRNA gene expression (Gali Ramamoorthy et al., 2015).

## Conclusion

This study highlights opportunities for the livestock industries to harness maternal dietary supplementation to alter progeny feed intake, enhance muscling, and alter fat deposition which in turn, may lead to an improvement in efficiency of meat production in beef cattle. This study provides evidence that protein supplementation during the periconception period and first trimester may alter offspring appetite and carcass traits. An improved understanding of the mechanisms that regulate appetite and fetal cell lineage commitment into myocytes, adipocytes, or fibroblasts in the bovine is required, and additional studies utilising larger experimental cohorts are warranted.

## Data Availability Statement

The original contributions presented in the study are included in the article/Supplementary Material, further inquiries can be directed to the corresponding author.

## Ethics Statement

The animal study was reviewed and approved by University of South Australia IMVS Animal Ethics Committee (Australia), The University of Adelaide (Australia), and The University of New England (Australia) Animal Ethics Committees (Approval numbers: 18/11, S2012-249 and AEC14-037, respectively). Written informed consent was obtained from the owners for the participation of their animals in this study.

## Author Contributions

VP, IM, RR, BM, and KC contributed to conception and design of the study. KC, BM, MC, JG, GG, MV, RR, and VP contributed to the acquisition of data. KC and VP performed the statistical analysis. KC wrote the first draft of the manuscript. KC, BM, RR, and VP wrote the sections of the manuscript. All authors contributed to the article and approved the submitted version.

## Conflict of Interest

MC was employed by company Ridley Agriproducts, Australia.

The remaining authors declare that the research was conducted in the absence of any commercial or financial relationships that could be construed as a potential conflict of interest.

## Publisher’s Note

All claims expressed in this article are solely those of the authors and do not necessarily represent those of their affiliated organizations, or those of the publisher, the editors and the reviewers. Any product that may be evaluated in this article, or claim that may be made by its manufacturer, is not guaranteed or endorsed by the publisher.
