# Supplementary material for: Periconception and First Trimester Diet Modifies Appetite, Hypothalamic Gene Expression, and Carcass Traits in Bulls
Source: Front Genet. 2021 Sep 3;12:720242. doi: 10.3389/fgene.2021.720242 (PMC8448419; doi:10.3389/fgene.2021.720242)
Supplement: Supplementary file 1 [file Table_1.DOCX]

**Supplementary Table S1**. Ingredients and nutrient content of heifer rations for induction period, the PERI (-60 to 23 dpc) and POST-conception period (24 to 98 dpc) and 2nd and 3rd trimester of gestation (99 dpc to term)*.

|  | Induction | PERI  conception | | POST  conception | | 2nd and 3rd Trimester |
| --- | --- | --- | --- | --- | --- | --- |
|  |  | L | H | L | H |  |
| Ration as fed |  |  |  |  |  |  |
| Wheat (kg) | 0.66 | 1.81 | 0.48 | 2.12 | 0.56 | 0.60 |
| Canola meal (kg) | 2.23 | - | - | - | - | 0.89 |
| Soybean meal (kg) |  | 0.48 | 1.83 | 0.56 | 2.14 | 0.44 |
| Barley Straw (kg) | 7^1^ | 5.5 | 6.7 | 10.2 | 10.7 | 8.6 |
| Molasses (g) | 90 | 72 | 72 | 84 | 84 | 60 |
| Biofos MDCP (g) | - | 19 | - | 22 | - | - |
| Salt (g) | 15 | 12 | 12 | 14 | 14 | 10 |
| Vitamin / trace mineral Premix (g) | 3 | 2 | 2 | 3 | 3 | 2 |
| Dry Matter (kg) | 9.1^2^ | 7.2 | 8.3 | 11.8 | 12.3 | 9.6 |
| Total energy (MJ ME) |  | 63 | 71 | 98 | 102 | 79 |
| % of energy requirements^3^ |  | 85 | 96 | 136 | 142 | 125 |
| Total crude protein (kg) |  | 0.62 | 1.18 | 0.88 | 1.49 | 0.92 |
| % of protein requirements^3^ |  | 67 | 127 | 72 | 123 | 88 |
| % CP (total diet) |  | 8.6 | 14.2 | 7.4 | 12.1 | 9.6 |
| % Fat^2^ |  | 1.5 | 1.4 | 1.4 | 1.4 | 1.5 |
| % Starch^2^ |  | 15.1 | 4.7 | 10.9 | 3.8 | 4.8 |
| Total calcium (g) |  | 22 | 26 | 37 | 38 | 33 |
| % of calcium requirements^3^ |  | 110 | 130 | 185 | 190 | 132 |
| Total phosphorus (g) |  | 17 | 17 | 21 | 21 | 20 |
| % of phosphorus requirements^3^ |  | 130 | 130 | 160 | 160 | 125 |
|  |  |  |  |  |  |  |

^1^assumed value

^2^predicted value

^3^Dietary requirements were calculated using Nutrient Requirements of Domesticated Ruminants (Freer, 2007).

Input values were based upon nutrient analysis of component ingredients in the total diet, liveweight and age of heifers at each diet change, mature cow weight of 550 kg and the desired growth target.

L = Low H = High

Key assumptions:

Calculations use the formulated values for pellets and actual values for straw.

PERI-conception diet is based upon 340 kg Santa Gertrudis heifer gaining 0.5 kg/day.

POST-conception diet is based upon 400 kg, 60 dpc Santa Gertrudis heifer gaining 0.5 kg/day.

Second and 3rd trimester diet is based upon 480 kg, 200 dpc Santa Gertrudis heifer gaining 0.5 kg/day.

* Table S1 from Copping et al. (2018). Open Access, Creative Commons. <https://doi.org/10.1071/RD17102>

LITERATURE CITED

Copping, K.J., Ruiz-Diaz, M.D., Rutland, C.S., Mongan, N.P., Callaghan, M.J., McMillen, I.C., et al. (2018). Peri-conception and first trimester diet modifies reproductive development in bulls. *Reprod Fertil Dev* 30(5)**,** 703-720. doi: 10.1071/RD17102.

Freer, M. (2007). *Nutrient requirements of domesticated ruminants* Collingwood, Melbourne, Vic: CSIRO Publishing.
